# Editorial

**DOI:** 10.1002/osp4.68

**Published:** 2016-09-19

**Authors:** David B. Sarwer

**Affiliations:** ^1^ Obesity Science & Practice

I am excited to write to all of you with an update on *Obesity Science & Practice*. Summer is winding down, and football season has kicked off. While watching football is one of my favorite fall weekend pastimes, my beloved Chicago Cubs have the best record in baseball and are positioned to end a century of frustration and heartbreak for millions of long suffering fans throughout the world (no jinx, no curse of the Billy Goat here). That is not, however, the reason why I am excited to write this editorial. With the fourth issue of *Obesity Science & Practice* released in June 2016, we published our 25th article. This allows us to submit for inclusion in PubMed Central, which will further enhance the reach of the articles published in journal. The Wiley team has told me that, for an open access journal, we have reached this milestone sooner than most. I am pleased with that and thank our authors, the editors from the other Wiley obesity journals – *Obesity, Obesity Reviews, Pediatric Obesity and Clinical Obesity* – and the entire *Obesity Science & Practice* team for their efforts. This is a great accomplishment and one that I look for us to build upon going forward. I will keep you updated on progress with the process.

The present issue represents our fifth issue of the journal and third issue in 2016. Like the previous four issues, this issue includes studies of children and adults as well as papers focusing on issues of aetiology as well as treatment. Several the articles come from internationally known research groups and institutions; others come from newer investigators and teams who are already showing their scientific creativity and writing skills. These articles come to the journal in a number of ways. The other Wiley obesity journals refer articles to us with increasing regularity. At the same time, our direct submissions continue at a consistent pace. As a result, we currently have our greatest number of articles circulating through submission website. I am optimistic that these trends will continue, and we will be well positioned to release another issue before the end of the year.

Like many of you, I am looking forward to Obesity Week in New Orleans in a few months. As a Tulane alumnus, I am always excited to return to the city that I called home for 4 years and to visit places old and new. My early read on the programme suggests that Obesity Week will again be jam packed with outstanding presentations from researchers and thought leaders from across the globe as well as young investigators and students with tremendous potential to be the field's next leaders. As you start to work on your presentations and posters, I encourage you to think of *Obesity Science & Practice* as an outlet for the research you present at the meeting. For early career investigators, the journal is a great opportunity to showcase your work and help build your curriculum vitae. For established researchers, use the energy boost that comes from a stimulating scientific meeting to quickly turn your presentation into a manuscript for the journal before 2016 comes to a close. I am working closely with the Editorial Board to review and act on submitted manuscripts within 4 weeks and to move them through the publication process quickly. As a result, the manuscript from the work you present in New Orleans has the potential to be published in the journal in early 2017.

I look forward to seeing you in New Orleans. During the day, I plan to spend my time moving between the oral presentations, panel discussions and poster sessions. At night, if all goes well, you likely will find me not on Bourbon Street, but in a sports bar watching my Cubs in the World Series!

Le Bon Temps Roule and Go Cubs Go!

